# Heat and Dehydration Additively Enhance Cardiovascular Outcomes following Orthostatically-Stressful Calisthenics Exercise

**DOI:** 10.3389/fphys.2017.00756

**Published:** 2017-10-09

**Authors:** Ashley P. Akerman, Samuel J. E. Lucas, Rajesh Katare, James D. Cotter

**Affiliations:** ^1^School of Physical Education, Sport and Exercise Sciences, Division of Sciences, University of Otago, Dunedin, New Zealand; ^2^Department of Physiology, Division of Health Sciences, University of Otago, Dunedin, New Zealand; ^3^School of Sport, Exercise and Rehabilitation Sciences, University of Birmingham, Birmingham, United Kingdom

**Keywords:** hypotension, hypervolemia, aldosterone, erythropoietin, calisthenics, adaptation, orthostasis, hypohydration

## Abstract

Exercise and exogenous heat each stimulate multiple adaptations, but their roles are not well delineated, and that of the related stressor, dehydration, is largely unknown. While severe and prolonged hypohydration potentially “silences” the long-term heat acclimated phenotype, mild and transient dehydration may enhance cardiovascular and fluid-regulatory adaptations. We tested the hypothesis that exogenous heat stress and dehydration additively potentiate acute (24 h) cardiovascular and hematological outcomes following exercise. In a randomized crossover study, 10 physically-active volunteers (mean ± SD: 173 ± 11 cm; 72.1 ± 11.5 kg; 24 ± 3 year; 6 females) completed three trials of 90-min orthostatically-stressful calisthenics, in: (i) temperate conditions (22°C, 50% rh, no airflow; CON); (ii) heat (40°C, 60% rh) whilst euhydrated (HEAT), and (iii) heat with dehydration (no fluid ~16 h before and during exercise; HEAT+DEHY). Using linear mixed effects model analyses, core temperature (T_CORE_) rose 0.7°C more in HEAT than CON (95% CL: [0.5, 0.9]; *p* < 0.001), and another 0.4°C in HEAT+DEHY ([0.2, 0.5]; *p* < 0.001, vs. HEAT). Skin temperature also rose 1.2°C more in HEAT than CON ([0.6, 1.8]; *p* < 0.001), and similarly to HEAT+DEHY (*p* = 0.922 vs. HEAT). Peak heart rate was 40 b·min^−1^ higher in HEAT than in CON ([28, 51]; *p* < 0.001), and another 15 b·min^−1^ higher in HEAT+DEHY ([3, 27]; *p* = 0.011, vs. HEAT). Mean arterial pressure at 24-h recovery was not consistently below baseline after CON or HEAT (*p* ≥ 0.452), but was reduced 4 ± 1 mm Hg after HEAT+DEHY ([0, 8]; *p* = 0.020 vs. baseline). Plasma volume at 24 h after exercise increased in all trials; the 7% increase in HEAT was not reliably more than in CON (5%; *p* = 0.335), but was an additional 4% larger after HEAT+DEHY ([1, 8]; *p* = 0.005 vs. HEAT). Pooled-trial correlational analysis showed the rise in T_CORE_ predicted the hypotension (*r* = −0.4) and plasma volume expansion (*r* = 0.6) at 24 h, with more hypotension reflecting more plasma expansion (*r* = −0.5). In conclusion, transient dehydration with heat potentiates short-term (24-h) hematological (hypervolemic) and cardiovascular (hypotensive) outcomes following calisthenics.

## Introduction

Exercise is a multi-factorial, pluripotent stress that produces strain in several physiological systems. The nature of the stress dictates the profile of strain incurred, and thus the adaptive stimulus. The adaptations that occur as a result of this strain profile accrue as fitness, which improves an individual's physiological and functional capacities, and quality of life. The necessary stressors within exercise that are required to elicit a given response, or adaptation, are only partially resolved. Often the complex nature of exercise makes the delineation of these stressors difficult, and thus their individual and combined roles in adaptation also remain unclear.

Changes in blood pressure (BP) and blood volume (including plasma volume; PV) are principal components of the cardiovascular benefit of regular physical activity. However, the optimal means of facilitating these adaptations is elusive. The anti-hypertensive benefits associated with exercise (Pescatello et al., 2004, 2015) might be partly facilitated by repeated bouts of acute post-exercise hypotension (PEH; Thompson et al., 2001), and contribute to subsequent PV expansion (i.e., PVE/hypervolemia; Nagashima et al., 1999, 2000; Hayes et al., 2000). Assessing the acute cardiovascular outcomes to a conditioning stimulus, which are also important in their own right, may therefore also provide an indication of long-term cardiovascular adaptations (Liu et al., 2012). Exercise intensity moderates both PEH (Eicher et al., 2010; Halliwill et al., 2013; Graham et al., 2016) and PVE (Nadel et al., 1979; Convertino et al., 1981), such that a higher intensity elicits larger PEH and PVE, however finding an appropriate and effective exercise intensity to afford these benefits to all individuals is convoluted. Indeed, low-intensity exercise may be insufficient to confer these benefits (Wallace et al., 1999; Forjaz et al., 2004; Farinatti et al., 2011) despite its accessibility to the population. Thus, complementing low-intensity exercise with additional independent stressors may exacerbate the physiological strain, and provide an avenue for acute cardiovascular outcomes.

Heat stress and dehydration are by-products of exercise and stressors in their own right. The imposition of heat (Francesconi et al., 1983a; Ray and Gracey, 1997; Wright et al., 2010) and dehydration (Finberg et al., 1974; Nadel et al., 1980; Francesconi et al., 1983b; Brandenberger et al., 1986) increases strain in multiple systems at rest and in exercise, particularly when there is concurrent orthostatic stress (Beetham and Buskirk, 1958; Saltin and Stenberg, 1964; Ray et al., 1993; Gonzalez-Alonso et al., 1999). An increase in strain, and potential augmented adaptive stimulus, occurs despite low exercise intensity in dynamic aerobic (Finberg et al., 1974; Francesconi et al., 1983b), and isometric exercise (Ray and Gracey, 1997; Binder et al., 2012). Orthostasis and heat appear to be important stressors in stimulating PVE (Convertino et al., 1981; Nagashima et al., 1999, 2000) and potentially PEH (Halliwill et al., 2013), however the role of dehydration is equivocal and largely unexplored (Akerman et al., 2016). Considering that dehydration exacerbates the cardiovascular and hormonal (especially sympathetic and fluid regulatory) pathways associated with both PVE and PEH (Melin et al., 1997, 2001; Gonzalez-Alonso et al., 1999), it may also moderate these outcomes somewhat. Thus, identifying the separate and combined roles of these exercise- and environment-related stressors in driving or impairing adaptive processes is important for basic understanding and may aid in exercise prescription for a range of populations; especially those unable (or unwilling) to undertake moderate- and higher-intensity exercise.

The aim of the current study was therefore to determine the cumulative effects of orthostatically stressful, low-intensity exercise (dynamic body weight exercises, i.e., calisthenics), exogenous heat stress, and dehydration, on acute (24-h) cardiovascular and fluid regulatory outcomes. The separate effects of these stressors were not delineated *per se*, but rather their cumulative effects were examined in forming a composite stimulus (i.e., low-intensity exercise + heat stress + dehydration). This exercise type was chosen as an accessible mode and intensity for a majority of the population, whilst providing substantial cardiovascular strain, and force through all major muscle groups. We hypothesized that the addition of each stressor would provide a further stimulus for acute cardiovascular outcomes. Namely, plasma volume expansion at 24 h after exercise would be larger with the addition of each stressor, by virtue of inducing additional thermal and cardiovascular strain.

## Methods

### Participants

Ten habitually-active, young healthy individuals participated; six females and four males (mean ± SD, aged 24 ± 3 year, mass 72.1 ± 11.5 kg, and height 173 ± 11 cm). All were non-smokers, recreationally physically active on 3 to 5 days per week, and unacclimatised to heat. They were required to refrain from alcohol, caffeine and physical activity for 24 h before each experimental trial. All female participants had been taking the oral contraceptive pill for at least the previous 2 years, and undertook experimental trials in their self-reported early follicular phase. Written informed consent was provided by all individuals prior to participation, and all procedures were approved by the Institutional Human Ethics Committee of the University of Otago (Project No. 12/125), and conformed to the *Declaration of Helsinki*.

### Experimental protocol

#### Design

**Figure 1:**
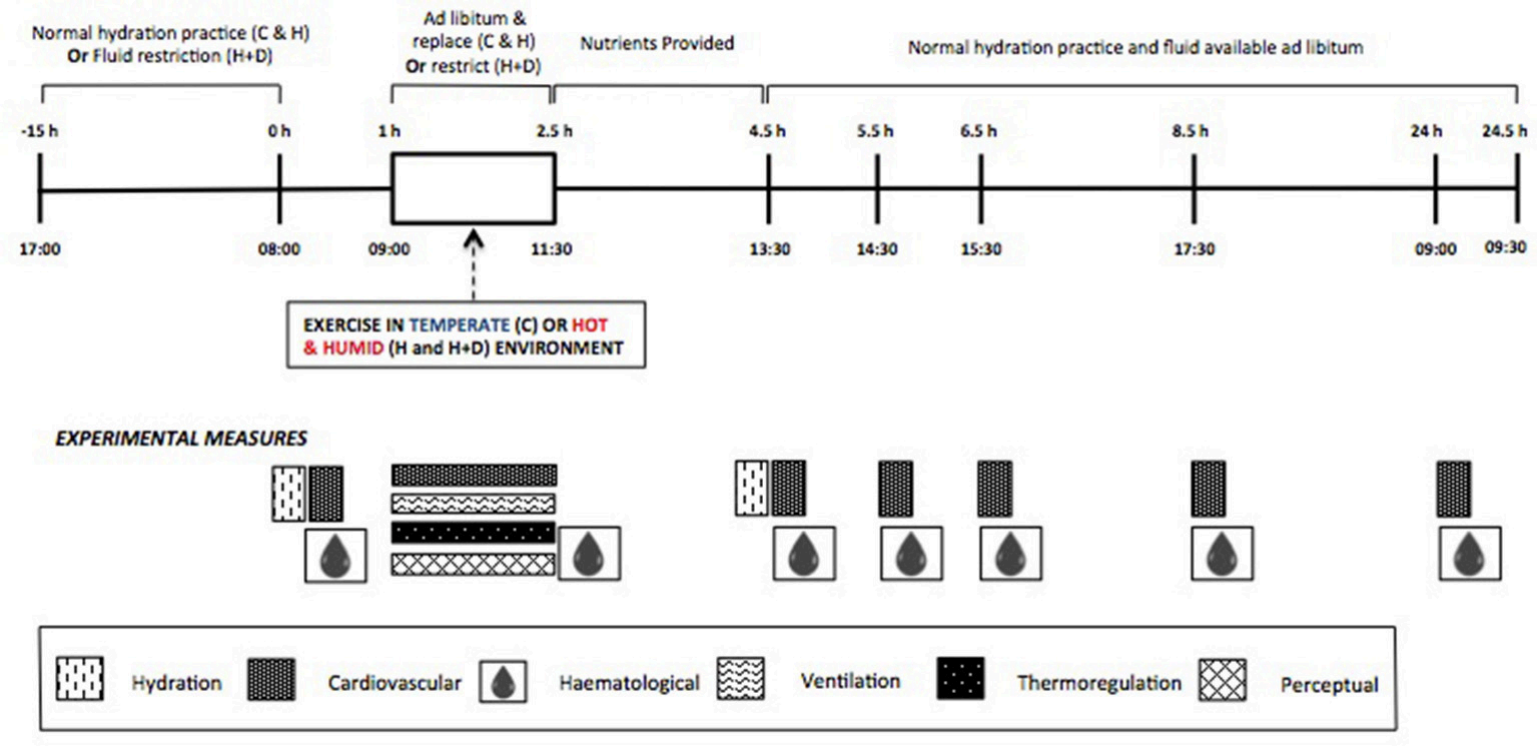
Schematic representation of standardizations and experimental measurements in the three exercise conditions (C, Control; H, Heat; H+D, Heat and dehydration).

The study was a randomized, counter-balanced crossover design. Following familiarization to the exercise protocol (one occasion in CON conditions, approximately 1 week prior to first exercise condition) and all experimental procedures and instrumentations, participants reported to the laboratory for three experimental trials, or “exercise conditions.” The three exercise conditions were: calisthenics in a temperate environment [22.2 ± 0.3°C, 51.2 ± 1.5% relative humidity (RH)] in a euhydrated state (i.e., CON); calisthenics in a hot, humid environment (40.4 ± 0.4°C, 60.5 ± 1.9% RH) in a euhydrated state (HEAT); and calisthenics in a hot, humid environment in a mildly-hypohydrated state, with further dehydration during the session (HEAT+DEHY). No exogenous airflow was provided. Each condition was at the same time of day (08:00 h) and included baseline measurements, a 90-min exercise protocol, and four follow-up measurement periods over 24 h of recovery (2, 3, 5, and 24 h after exercise; Figure 1). Exercise conditions were separated by 2–7 days, with a minimum of 2 days separating a temperate to hot condition, and 4 days between hot conditions. Participants recorded physical activity, food and fluid intake, and menstrual cycle logs for the 24 h prior to, and throughout the first exercise condition, and then replicated this for the remaining two conditions.

The three exercise conditions were completed on days that required similar 24-h physical activity requirements and often similar duration in the same posture (i.e., walking to and from appointments, and sitting in meetings or lectures). Participants were encouraged to consume 500 mL of water with dinner the night before, and another 500 mL with breakfast on the morning of the euhydrated conditions (CON and HEAT). Further to *ad libitum* fluid intake (water) during the euhydrated trials, body mass (BM) was measured at 15-min intervals during exercise, and sufficient water was provided to replace any BM deficit. The dehydration protocol in HEAT+DEHY consisted of ~16-h fluid restriction (from 17:00 h the night before, to 09:00 h on day of testing; i.e., hypohydration), followed by no fluid replacement during the exercise.

#### Procedure

Participants arrived at the laboratory following a (self-reported) standardized fluid and meal intake. After voiding their bladder (second void of the morning) and inserting a rectal thermistor, participants were weighed semi-nude (underwear only) then rested supine before blood pressure (BP) was recorded and venous blood was drawn. Participants were then seated for 10 min to stabilize fluid compartment volumes (Ahlgrim et al., 2010) before capillary blood samples were obtained for tracking changes in plasma volume (PV). During this rest period participants were also instrumented with skin thermistors and a heart rate (HR) monitor.

Participants then entered an environmental chamber and sat for 5 min before recording of their resting HR, core (T_CORE_) and skin temperatures (T_SKIN_), and psychophysical perceptions (detailed below). This 5-min period was replicated at the end of the protocol. Participants then completed 10 min of squats at 0.2 Hz (5 min at start and end), and 70 min of calisthenics. Thus, the total “exercise protocol” was 90-min, consisting of the seated rest period, squats, and calisthenics. Respiratory gases, thermal, cardiac, and perceived strain was recorded throughout. The squat procedure was intended to provide insight into cerebrovascular autoregulation following orthostatically stressful exercise. Due to technical problems maintaining cerebrovascular measures throughout trials in the heat, these data are not of sufficient quality to present.

Participants then left the environmental chamber and sat in a temperate environment (~26°C, 27% RH) for 10 min before capillary blood samples were obtained, and lay supine for another 10 min before BP was measured and another venous blood sample was drawn (both supine). Participants voided their bladder, toweled dry and had semi-nude body mass measured. Participants were then provided with 250 mL of yogurt (37 g carbohydrate, 9 g protein, 4 g fat and 90 mg sodium) and 500 mL of fruit juice (1:1 fruit juice and water mix, 25 g carbohydrate, 1 g protein, and ~820 mg sodium) to consume as soon as possible, and return to their normal hydration level *ad libitum* thereafter (same juice/salt/water mix available throughout recovery). This standardized meal was chosen due to the importance of immediate protein (De Feo et al., 1992) and sodium (Luetkemeier et al., 1997) supplementation on outcome measures.

Participants returned to the laboratory 2, 3, 5, and 24 h after completing the exercise protocol for recovery measures. Participants provided a urine sample, and semi-nude body mass measurement, followed by BP measurement, venous blood (both supine after 10-min rest), and capillary blood samples after 10 min of seated rest (Figure 1).

#### Exercise protocol

The exercise protocol was intended to represent a routine suitable for individuals unable to complete more traditional exercise forms. The composition and progression of exercises was designed specifically for this study, with particular emphasis on a whole-body stress and repeated changes in posture (i.e., orthostatically stressful). A detailed description of the calisthenics protocol is provided in Supplementary Data Sheet 1 (https://www.youtube.com/watch?v=FxMdPDUk8Fo) and Supplementary Figure 1. To summarize, 28 body-weight resistance movements (i.e., calisthenics), and yoga postures (herein referred to collectively as “exercises”) were completed for 70 min. The protocol consisted of ~35 min of standing exercises (7 exercises at start, 5 exercises at the end), ~15 min of supine exercises (7 exercises), ~15 min of exercises on hands and knees (6 exercises), and ~5 min of prone exercises (3 exercises).

### Measurements

Semi-nude body mass was measured to a resolution of 20 g using calibrated electronic scales (Wedderburn, Dunedin, New Zealand) before and after each exercise condition, and at 15-min intervals during the two hydrated exercise conditions (i.e., CON and HEAT). Specific gravity of urine (U_SG_) was measured using a hand-held refractometer (Atago, Tokyo, Japan) before and after exercise.

Blood pressure was measured using a stethoscope and sphygmomanometer, by the same researcher (AA) on all occasions, and reported as the mean of 3 readings over ~5 min. Heart rate was measured from the R-R interval of ventricular depolarisation, and recorded at 1-min intervals via telemetry to a wristwatch (Model RS400, Polar, Finland). Core temperature was measured using a rectal thermistor (400 series; Mallinckrodt Inc. USA) inserted 10 cm beyond the external anal sphincter. Skin temperature was measured using thermistors attached to the thigh, chest, back, and bicep using adhesive tape. Core and skin temperatures were recorded at 1-min intervals (DaqPRO 5300, Omega, USA.). Ratings of perceived exertion (RPE, ranging from 6 to 20; Borg, 1982), thermal sensation (ranging from 1 to 13), and thermal discomfort (ranging from 1 to 5; both extended from Gagge et al., 1967) were obtained using 15-, 13-, and 5-point scales respectively. Expired oxygen (O_2_) and carbon dioxide (CO_2_) concentrations and volumes were determined using open-circuit spirometry (Cosmed, CosmedSrl, Rome, Italy) to calculate oxygen consumption ($\overset{\cdot }{\text{V}}$O_2_) and partial pressure of end-tidal carbon dioxide (P_ET_CO_2_). Gas analysers were calibrated before a participant's exercise session.

Arterialised capillary blood samples (75 μL) were obtained from the finger, in triplicate. Hemoglobin (Hb) concentrations were determined using reflectance photometry (OSM3; Radiometer, Copenhagen, Denmark). Hematocrit (Hct) was measured from the remaining portion (~50 μL) of each sample using a custom-made Vernier caliper reader after centrifugation (Thermo IEC, MicroCL 17, radius 8.5 cm) for 10 min at 855 g.

Venous blood samples (18 mL) were drawn without stasis from an antecubital vein. Whole-blood samples were centrifuged immediately at 4°C for 10 min, and plasma was separated and stored at −80°C for subsequent batch analysis. Plasma osmolality (P_OSM_) was measured using a three-point calibrated (100, 290, and 1,000 mOsmol·kg^−1^) dew-point temperature depression method, in duplicate (triplicate if ≥2 mOsmol·kg^−1^ difference; Model 5,520, Vapro osmometer, Wescore, Logan, UT, USA). Plasma aldosterone (P_ALD_) and erythropoietin (P_EPO_) were measured using enzyme-linked immunosorbent assay (ELISA) commercially available kits (Abnova, Taiwan; and Abcam, Australia, respectively). All samples for a given participant were analyzed within the same assay, and handled by the same researcher (AA or RK).

Plasma aldosterone concentrations were determined from calibration standards (0–1,000 pg·mL^−1^) and control samples, using a standard curve derived from the log transformation of the optical density of each sample. The curve was generated using commercially available software (GraphPad Prism, Version 6, GraphPad Software, CA, USA). The intra-assay coefficient of variation for P_ALD_ concentration, derived from the calibration standards, was 14.4%. Plasma erythropoietin sample concentrations were determined from calibration standards (0–100 mIU·mL^−1^) and control samples, using an applied four-parameter algorithm fit using a free internet-based ELISA analysis program (elisaanalysis.com). The intra-assay coefficient of variation for P_EPO_ concentration, derived from the calibration standards, was 5.3%.

### Data analysis

#### Calculations and derivations

All measurements are *n* = 10 unless stated otherwise. To describe the effect of the dehydration protocol (i.e., vs. normal hydration/euhydration), an unstressed baseline was estimated by averaging CON and HEAT baseline measurements, against which variables are compared over time where relevant (i.e., ΔBM, ΔBP, and ΔPV).

Area-weighted T_SKIN_ was calculated according to International Organisation for Standardization (1992) from the following formula:

$$\begin{array}{l} {\text{T}}_{\text{SKIN}}=((0.175^{*}{\text{T}}_{\text{CHEST}})+(0.175^{*}{\text{T}}_{\text{BACK}})\\ \;+(0.19^{*}{\text{T}}_{\text{BICEP}})+(0.39^{*}{\text{T}}_{\text{THIGH}}))/0.93 \end{array}$$

Changes in PV, associated with the exercise conditions, were calculated from changes in Hb and Hct according to the equation of Strauss et al. (1952):

$$\begin{array}{l} \%\Delta \text{PV}=100\;[({\text{Hb}}_{0}/{\text{Hb}}_{\text{t}}{)}^{*}((1-{\text{Hct}}_{\text{t}})/(1-{\text{Hct}}_{0}))]-100 \end{array}$$

where t and 0 subscripts denote measurements at time t and baseline (unstressed), respectively. Hb is in g·100 mL^−1^ and Hct is a fraction.

#### Statistics

Variables were individually analyzed longitudinally using linear mixed effect model analysis. Where appropriate, models incorporated up to seven repeated measurements (i.e., average hydrated, baseline, immediately post-exercise, 2-, 3-, 5-, and 24-h post-exercise) across three separate conditions (CON, HEAT, HEAT+DEHY). The order of condition completion was incorporated as a fixed effect, while participant was modeled as a random effect. Homogeneity of variances was assessed visually via plotting of residuals vs. model-fitted values, and formally with Levene's test, across all combinations of factors in the model. Linearity and approximate normal distribution of residuals were assessed via visual inspection of histograms and Q-Q plots of model and individual residuals, and formally with Shapiro-Wilk test. Akaike' Information Criteria (AIC) was used to determine covariance structure of model errors (following significant Levene's test), random-effect structure, and incorporation of fixed effects. In the presence of non-significant (via AIC) condition:time interaction, and thus non-inclusion in the model, “NS” is reported. Multiple comparisons were made using the least squares means contracts derived from the mixed models, and adjustments for multiple testing made with the Hochberg-Bonferroni method. In line with the study aims and hypotheses being focused on delineating the addition of discrete stressors, only comparisons between conditions of interest (i.e., HEAT vs. CON, and HEAT+DEHY vs. HEAT) were examined.

Associations between mechanistically related variables were explored using standard least squares regressions. The relations between the variables are expressed as the Pearson's correlation coefficient (denoted as *r*) and corresponding *p*-values. The magnitude of correlation effect was qualitatively assessed from corresponding according to Hopkins et al. (2009); trivial (*r* < 0.1), small (0.1 < *r* < 0.3), moderate (0.3 < *r* < 0.5), large/strong (0.5 < *r* < 0.7), and very large/strong (0.7 < *r* < 0.9).

The mixed-model analysis was performed using R (R Development Core Team, 2008), and correlations were performed on raw values using GraphPad Prism (Prism Version 7.00, GraphPad Software, California, USA). Descriptive statistics in text are reported as raw means ± SD, whereas comparisons of interest are reported as least-squares means with corresponding 95% confidence limits [lower limit, upper limit]. A *p* < 0.05 was considered statistically significant *a priori*. To aid in interpretation, ANOVA-derived main effects and interactions are provided, and (where appropriate) only *post-hoc p*-values are presented in corresponding table or text.

## Results

Physical activity, diet, and menstrual cycle logs were reviewed prior to starting each of the second and third exercise conditions, and hydration compliance was checked verbally at this time. Prior to the two euhydrated conditions (CON and HEAT), if participants rated their thirst to be other than “not thirsty” (i.e., 1 on a 9-point validated Thirst Scale; Riebe et al., 1997), they ingested additional water *ad libitum*. All participants completed the 90-min exercise protocol.

### Hydration

#### Body mass changes (Δ BM)

When compared against an average hydrated body mass (72.2 ± 11.8 kg), the overnight fluid restriction in HEAT+DEHY resulted in 1.1 ± 0.8% BM reduction (71.3 ± 11.6 kg), with a further 1.8 ± 0.8% loss by the end of exercise, leading to a net loss of 2.9 ± 1.2% (70.0 ± 11.0 kg). Net mass losses were 0.5 ± 0.6% in CON (71.8 ± 11.8 kg), and 0.1 ± 0.6% in HEAT (71.9 ± 11.3 kg; *n* = 9; *p* = 0.527 vs. CON).

#### Urine specific gravity (U_SG_)

Baseline U_SG_ was similar between HEAT (1.007 ± 0.005) and CON (1.008 ± 0.006, *p* = 0.800), but was 0.019 higher in HEAT+DEHY (1.026 ± 0.005) than in HEAT (95% CL: [0.014, 0.023]; *p* < 0.001). Across exercise, U_SG_ was unchanged in CON (*p* = 0.155) and HEAT (*p* = 0.289), but tended to increase in HEAT+DEHY (*p* = 0.075). As such, post-exercise U_SG_ was similar between HEAT (1.005 ± 0.005) and CON (1.005 ± 0.004; *p* = 0.962), but 0.024 higher in HEAT+DEHY (1.029 ± 0.004) than in HEAT ([0.019, 0.028]; *p* < 0.001).

#### Plasma osmolality (P_OSM_; Figure 2)

**Figure 2:**
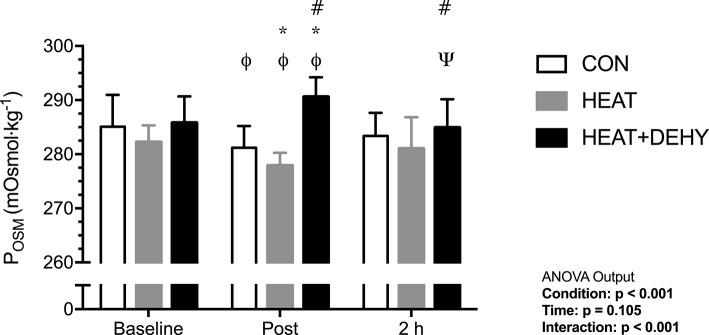
The additive effects of heat stress and hydration status on plasma osmolality (P_OSM_) before, immediately after, and 2 h after 90-min exercise in CON (all *n* = 10), HEAT (*n* = 9, 9, and 8 resp.), and HEAT + DEHY (*n* = 9, 9, and 10 resp.). Statistical significance (ANOVA Output) is illustrated. Data presented are group mean ± SD. ^Φ^Denotes significantly different (*p* < 0.05) to baseline. ^Ψ^Denotes significantly different to post-exercise measurement. ^*^Denotes significantly different than CON. ^#^Denotes significantly different to HEAT.

Baseline P_OSM_ was similar between HEAT and CON (*p* = 0.243), and HEAT and HEAT+DEHY (*p* = 0.111; Figure 2). Across exercise, P_OSM_ decreased by 4 and 5 mOsm·kg^−1^ in CON and HEAT, respectively, but increased by a further 4 mOsm·kg^−1^ in HEAT+DEHY (all *p* ≤ 0.014). As such, post-exercise P_OSM_ was lower in HEAT than in CON (mean difference: −4 mOsm·kg^−1^; [−6, −1]; *p* = 0.002), and higher in HEAT+DEHY than in HEAT (13 mOsm·kg^−1^; [10, 15]; *p* < 0.001). Across the first 2 h of recovery, P_OSM_ remained stable in CON (*p* = 0.249) and HEAT (*p* = 0.128), but decreased by 5 mOsm·kg^−1^ in HEAT+DEHY (*p* = 0.001), remaining 4 mOsm·kg^−1^ higher in HEAT+DEHY than in HEAT at 2 h ([0, 9]; *p* = 0.046).

### Thermal (Table 1)

Average core temperature (T_CORE_) was 0.4°C higher with the addition of heat stress, and an additional 0.2°C higher with dehydration (Table 1). Peak T_CORE_ was 0.7°C higher with the addition of heat stress, and 0.3°C higher with dehydration (Table 1). Mean skin temperature (T_SKIN_) was 5.0°C warmer with the addition of heat stress, but not additionally higher with dehydration (Table 1). Peak T_SKIN_ however was 5.4°C warmer with the addition of heat stress, and an additional 0.2°C warmer with dehydration (Table 1).

**Table 1:** The additive effects of heat stress and hydration status on the average and peak thermal, cardiac and perceptual strain during the 90-min exercise condition, with corresponding statistical significance (ANOVA Output) and comparisons of interest.

	**Exercise condition**			***P*-value**	***H–C***	***H+D–H***
	***C***	***H***	***H+D***		***Mean diff; [95% CL]; p-value***	
**THERMAL**						
Average T_CORE_ (°C)	37.2 ± 0.3	37.6 ± 0.3	37.7 ± 0.3	<0.001	0.4; [0.2, 0.6]; <0.001	0.2; [0.0, 0.4]; = 0.059
Peak T_CORE_ (°C)	37.3 ± 0.2	38.0 ± 0.3	38.3 ± 0.3	<0.001	0.7; [0.5, 0.8]; <0.001	0.3; [0.2, 0.5]; <0.001
Average T_SKIN_ (°C)	31.6 ± 0.6	36.7 ± 0.3	36.8 ± 0.2	<0.001	5.0; [4.5, 5.5]; <0.001	0.2; [−0.1, 0.4]; = 0.328
Peak T_SKIN_ (°C)	31.8 ± 0.6	37.2 ± 0.3	37.4 ± 0.2	<0.001	5.4; [4.9, 5.8]; <0.001	0.2; [0.0, 3.3]; = 0.025
**CARDIAC**						
Average HR (b·min^−1^)	86 ± 9	115 ± 13	126 ± 12	<0.001	31; [27, 35]; <0.001	11; [6, 15]; <0.001
Peak HR (b·min^−1^)	100 ± 9	139 ± 19	154 ± 14	<0.001	40; [28, 51]; <0.001	15; [3, 27]; = 0.011
**PERCEPTIONS**						
Peak RPE (AU)	9.6 ± 2.4	13.0 ± 2.5	15.3 ± 1.6	<0.001	2.5; [1.5, 3.6]; <0.001	3.2; [2.2, 4.2]; <0.001
Peak TS (AU)	6.9 ± 0.6	10.8 ± 1.7	12.0 ± 0.9	<0.001	3.9; [2.4, 5.3]; <0.001	1.2; [−0.5, 2.9]; = 0.176
Peak TD (AU)	1.0 ± 0.0	3.1 ± 1.2	4.3 ± 0.9	<0.001	2.1; [1.2, 2.9]; <0.001	1.2; [0.3, 2.0]; = 0.006

*Data presented are descriptive mean ± SD. All n = 10. Model estimates of differences between conditions of interest are presented as mean difference, (95% confidence limits), and corresponding p values (post-hoc). C, CON; H, HEAT; H+D, HEAT+DEHY. H–C, independent effect of heat. H+D–H, independent effect of dehydration. HR, heart rate. T_CORE_, core (rectal) temperature. T_SKIN_, mean skin temperature. RPE, Rating of perceived exertion; TS, Thermal sensation; TD, Thermal discomfort*.

### Heart rate (Table 1)

Average HR was 37% higher with the addition of heat stress to exercise (i.e., CON vs. HEAT), and an additional 10% higher with dehydration (i.e., HEAT vs. HEAT+DEHY; Table 1). Similarly, peak HR was 41% higher with heat stress and 11% higher with dehydration (Table 1).

### Perceptual responses (Table 1)

Participants perceived exercise to be additively harder with the imposition of heat stress and dehydration (Table 1). They also reported higher ratings of thermal sensation with the addition of heat stress, but not additionally with dehydration (Table 1), and an additively higher thermal discomfort with heat stress and dehydration (Table 1).

### Respiratory (Table 2)

Mean oxygen consumption ($\overset{\cdot }{\text{V}}$O_2_) and carbon dioxide production ($\overset{\cdot }{\text{V}}$CO_2_) were similar between conditions (Table 2), as were changes from baseline (all *p* ≥ 0.312; Table 2). Whereas, mean ventilation (*n* = 8) and its component factors (frequency and tidal volume) increased 51% more with the addition of heat stress, but not additionally with dehydration (Table 2). Mean end-tidal carbon dioxide pressure (P_ET_CO_2_; Table 2) were therefore lower, by 9%, with the addition of heat stress, but not additionally with dehydration (Table 2). By end exercise, the reduction in P_ET_CO_2_ was 74% larger with heat stress, but also not additionally with dehydration (Table 2).

**Table 2:** The additive effects of heat stress and hydration status on the average and change from baseline in respiratory strain during the 90-min exercise condition, with corresponding statistical significance (ANOVA Output) and comparisons of interest.

	**Exercise condition**			***P*-value**	***H–C***	***H+D–H***
	***C***	***H***	***H+D***		***Mean diff; [95% CL]; p-value***	
**RESPIRATORY**						
Average $\overset{\cdot }{\text{V}}$O_2_ (mL·min^−1^)	757 ± 177	754 ± 170	764 ± 177	0.847		
Δ $\overset{\cdot }{\text{V}}$O_2_ (mL·min^−1^)	323 ± 146	343 ± 146	370 ± 143	0.032	32; [−31, 94]; = 0.409	36; [−25, 97]; = 0.312
Average $\overset{\cdot }{\text{V}}$CO_2_ (mL·min^−1^)	638 ± 180	667 ± 181	669 ± 180	0.236		
Δ $\overset{\cdot }{\text{V}}$CO_2_ (mL·min^−1^)	266 ± 119	289 ± 129	280 ± 107	0.943		
Average P_ET_CO_2_ (mm Hg)	36 ± 3	32 ± 4	30 ± 4	<0.001	−3; [−1, −6]; = 0.013	−2; [−5, 0]; = 0.104
Δ P_ET_CO_2_ (mm Hg)	−4 ± 1	−7 ± 3	−11 ± 6	0.001	3; [0, 5]; = 0.020	4; [−1, 10]; = 0.154
Average V°_E_ (L·min^−1^)	18 ± 4	20 ± 3	21 ± 4	0.045	2.1; [−0.9, 5.1]; = 0.182	0.9; [−2.2, 3.9]; = 0.741
Δ V°_E_ (L·min^−1^)	7 ± 5	11 ± 7	12 ± 10	0.018	3; [0, 5]; = 0.019	−1; [−3, 2]; = 0.629
Average B_F_ (breaths·min^−1^)	21 ± 4	21 ± 4	22 ± 4	0.765		
Δ B_F_ (breaths·min^−1^)	9 ± 3	8 ± 4	9 ± 3	<0.001	3.3; [1.1, 5.5]; = 0.005	0.03; [−2.6, 2.0]; = 0.943
Average V°_T_ (L·min^−1^)	0.9 ± 0.2	0.9 ± 0.2	1.0 ± 0.2	0.112		
Δ V°_T_ (L·min^−1^)	0.6 ± 0.4	0.7 ± 0.5	0.6 ± 0.3	0.641		

*Data presented are descriptive mean ± SD. Model estimates of differences between conditions of interest are presented as mean difference, (95% confidence limits), and corresponding p-values (post-hoc). C, CON; H, HEAT; H+D, HEAT+DEHY. H–C, independent effect of heat. H+D–H, independent effect of dehydration. $\overset{\cdot }{\text{V}}$O_2_, oxygen consumption. $\overset{\cdot }{\text{V}}$CO_2_, carbon dioxide production. P_ET_CO_2_, end-tidal carbon dioxide. $\overset{\cdot }{\text{V}}$_E_, ventilation (n = 8). B_F_, breathing frequency (n = 8). $\overset{\cdot }{\text{V}}$_T_, tidal volume (n = 8)*.

### Blood pressure (BP; Table 3 and Figure 3)

**Figure 3:**
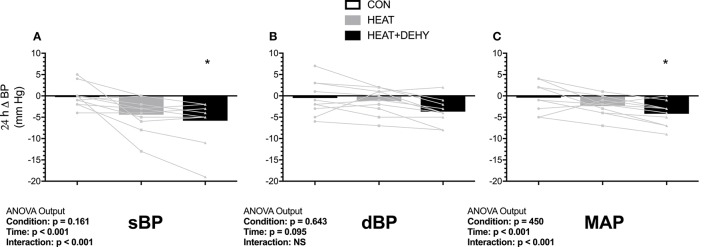
The additive effects of heat stress and hydration status on the 24-h change in systolic (sBP, **A**), diastolic (dBP, **B**) and mean arterial blood pressure (MAP, **C**) after the 90-min exercise condition. Statistical significance (ANOVA Output) is illustrated. Data presented are group mean with individual values overlaid. ^*^Denotes significantly different (*p* < 0.05) than CON.

Unstressed baseline sBP, dBP and MAP were 108 ± 6, 69 ± 7, and 82 ± 5 mm Hg, respectively. Diastolic blood pressure (dBP) did not change significantly, whereas systolic (sBP) and mean arterial pressure (MAP) showed reductions as described below (Table 3, Figure 3). Individual responses are provided in Supplementary Figure 2 for reference.

**Table 3:** The additive effects of heat stress and hydration status on arterial blood pressure before and after the 90-min exercise condition.

	**Baseline**			**Post-exercise**			**2-h post-exercise**			**3-h post-exercise**			**5-h post-exercise**			**24-h post-exercise**		
	***C***	***H***	***H+D***	***C***	***H***	***H+D***	***C***	***H***	***H+D***	***C***	***H***	***H+D***	***C***	***H***	***H+D***	***C***	***H***	***H+D***
sBP (mm Hg)	108 ± 6	107 ± 6	113 ± 6	108 ± 7	99 ± 5	108 ± 7	108 ± 7	109 ± 9	111 ± 8	107 ± 6	106 ± 7	107 ± 8	106 ± 7	105 ± 5	105 ± 7	107 ± 7	103 ± 5	102 ± 6
dBP (mm Hg)	68 ± 7	69 ± 8	71 ± 8	69 ± 7	70 ± 8	65 ± 8	69 ± 5	70 ± 6	70 ± 8	69 ± 5	67 ± 6	69 ± 5	68 ± 6	69 ± 7	68 ± 6	68 ± 6	67 ± 6	65 ± 6
MAP (mm Hg)	81 ± 5	82 ± 6	85 ± 6	82 ± 5	83 ± 7	77 ± 6	82 ± 3	83 ± 5	83 ± 6	81 ± 4	80 ± 5	81 ± 5	81 ± 5	81 ± 5	81 ± 4	81 ± 5	79 ± 5	77 ± 5

*Data presented are group mean ± SD. All n = 10. C, CON; H, HEAT; H+D, HEAT+DEHY. sBP, systolic blood pressure; dBP, diastolic blood pressure; MAP, mean arterial blood pressure. For statistical significance see Figure 3*.

In response to exercise, sBP remained unchanged in CON (all *p* ≥ 0.990 vs. baseline), and in HEAT (*p* ≥ 0.345) except tending to be reduced at 24-h after exercise (by 5 mm Hg [0, 11]; *p* = 0.077). Whereas, sBP was elevated by 6 mm Hg at baseline in HEAT+DEHY ([0, 11]; *p* = 0.033 vs. unstressed baseline), but 7 mm Hg lower immediately post-exercise ([2, 13]; *p* = 0.002), then unaffected at 2–5 h (all *p* ≥ 0.494), and reduced again by 5 mm Hg at 24 h ([0, 11]; *p* = 0.049). The MAP was unchanged from baseline in CON and HEAT at all time points (*p* ≥ 0.452), but in HEAT+DEHY it progressed from being equivalent at baseline (*p* = 0.206), to a 5 mm Hg reduction at end exercise ([1, 9]; *p* = 0.005), equivalent from 2 to 5 h (*p* ≥ 0.828), and reduced again by 4 mm Hg at 24 h ([0, 8]; *p* = 0.020).

When comparing between stressors at a given time, the addition of heat affected neither sBP nor MAP at end exercise (*p* ≥ 0.753 vs. CON), whereas the addition of dehydration reduced both; sBP by 7 mm Hg ([2, 11]; *p* < 0.001 vs. HEAT) and MAP by 6 mm Hg ([3, 9]; *p* < 0.001). The BP were thereafter unaffected by the addition of either stressor in the first 5 h of recovery (all *p* ≥ 0.219), whereas by 24 h, sBP tended to be reduced following HEAT (by 4 mm Hg; [0, 8]; *p* = 0.061), comparably to HEAT+DEHY (*p* = 0.837 vs. HEAT; Figure 3). At 24 h, MAP was similar in HEAT and CON (*p* = 0.218), and in HEAT+DEHY (*p* = 0.398 vs. HEAT).

#### Association between stress-induced strain and post-exercise hypotension (PEH; Figure 4)

**Figure 4:**
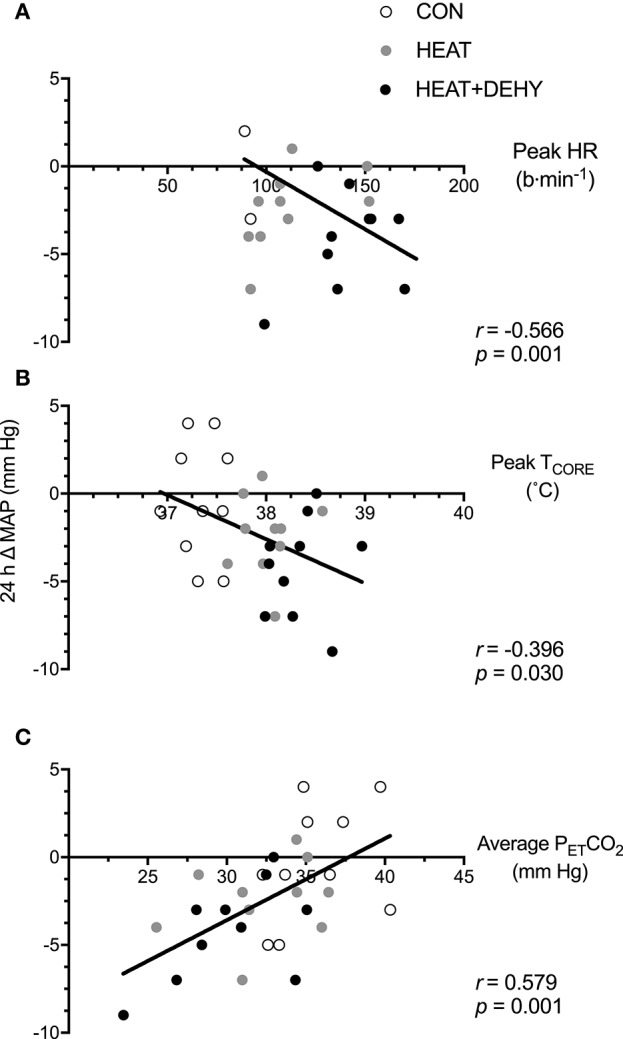
The relation between different forms of stress-induced strain and the post-exercise hypotensive (PEH) response at 24 h. Illustrated are the contributions of the cardiovascular strain (**A**; peak HR in exercise), thermoregulatory strain (**B**; peak T_CORE_ in exercise), and an indicator of respiratory strain (**C**; average end-tidal carbon dioxide (P_ET_CO_2_) in exercise) to the 24-h change in MAP.

The association between stress-induced strain and 24-h change in MAP were explored to examine whether the addition of individual stressors (i.e., exercise, heat, and dehydration) provided a further stimulus for adaptation, by virtue of each inducing greater strain. When data from the three conditions were pooled for correlational analysis, the strain during exercise was strongly associated with the magnitude of PEH at 24 h (Figure 4).

### Plasma volume (PV; Figure 5)

As per the analysis of BP, time effects were examined against an unstressed baseline (i.e., mean of both euhydrated conditions), whereas effects of heat and dehydration at a given time were examined by comparing their respective differences from this common baseline.

**Figure 5:**
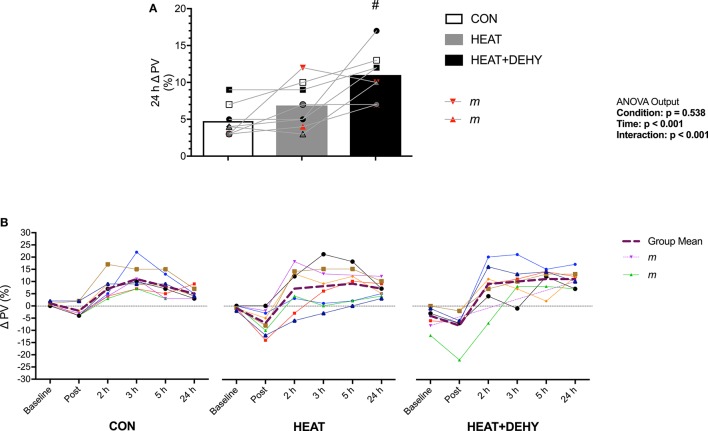
The additive effects of heat stress and hydration status on the change in plasma volume at 24 h after exercise (**A**; *n* = 8), and the pattern of response **(B)** vs. the average hydrated measure immediately after, 2, 3, 5, and 24 h after exercise in CON (all *n* = 8), HEAT (*n* = 8, 8, 8, 8, 7, and 8 resp.), and HEAT+DEHY (*n* = 8, 7, 7, 7, 7, and 8 resp.). Statistical significance (ANOVA Output) is illustrated. Data presented are group mean with individual values overlaid. Two male participants (“*m*”) are highlighted for reference. #Denotes significantly (*p* < 0.05) different to HEAT.

Plasma volume (PV) was reduced at baseline by 3% in HEAT+DEHY ([1, 6]; *p* = 0.016). Compared to baseline, PV was significantly reduced post-exercise in HEAT (*p* = 0.007), but not significantly in CON or DEHY (both *p* ≥ 0.401). This reduction was larger with heat ([0, 10]; *p* = 0.072), but not additionally with dehydration ([−1, 2]; *p* = 0.959). By 2 h after exercise PV recovered such that it was no different to baseline in CON (*p* = 0.114), but higher than baseline in HEAT (*p* = 0.049) and DEHY (*p* < 0.001). Plasma volume then remained elevated throughout recovery in all conditions (all *p* ≤ 0.017), and to a similar extent from 2 to 5 h after exercise (all *p* ≥ 0.420; Figure 5). However, at 24 h the PV expansion was similar between HEAT and CON ([−1, 5]; p = 0.335) but 4% larger in HEAT+DEHY ([1, 8]; *p* = 0.005 vs. HEAT).

#### Association between stress-induced strain and plasma volume expansion (PVE; Figure 6), and between PEH and PVE (Figure 7)

**Figure 6:**
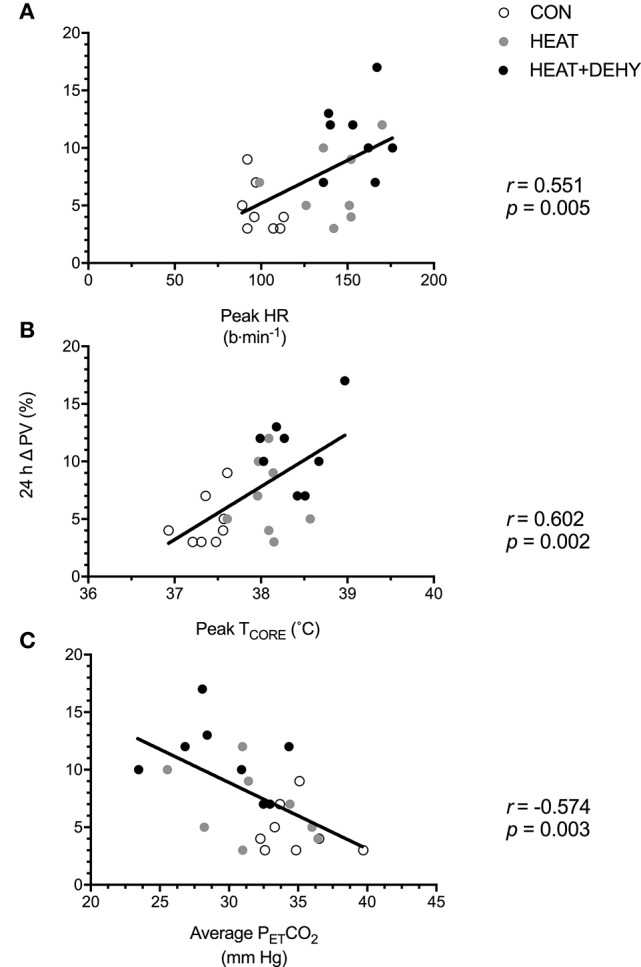
The relation between different forms of stress-induced strain and the post-exercise hypervolemic response (PVE; *n* = 8). Illustrated are the contributions of the cardiovascular strain (**A**; peak HR in exercise), thermoregulatory strain (**B**; peak T_CORE_ in exercise), and an indicator of respiratory strain (**C**; average P_ET_CO_2_ in exercise) to the 24-h change in PV.

**Figure 7:**
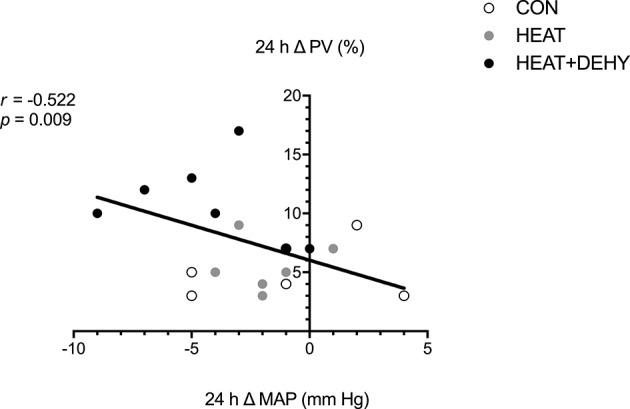
The relation between the post-exercise hypotensive response (24-h change in MAP), and the hypervolemic response (24-h change in PV; *n* = 8).

The association between indices of strain during exercise and expansion of PV by 24 h after exercise was explored to examine whether the addition of individual stressors (i.e., exercise, heat, and dehydration) provided a further stimulus for adaptation, by virtue of causing cumulative strain. Furthermore, the association between PEH and PVE (both at 24 h) was explored to examine potential mechanisms of exercise-induced hypervolemia (Figure 7). Cumulative strain was strongly associated with the magnitude of PVE at 24 h (Figure 6). Similarly, PVE at 24 h was strongly predictive of PEH at 24 h (Figure 7).

### Hormones

#### Aldosterone (ALD; Figure 8A)

**Figure 8:**
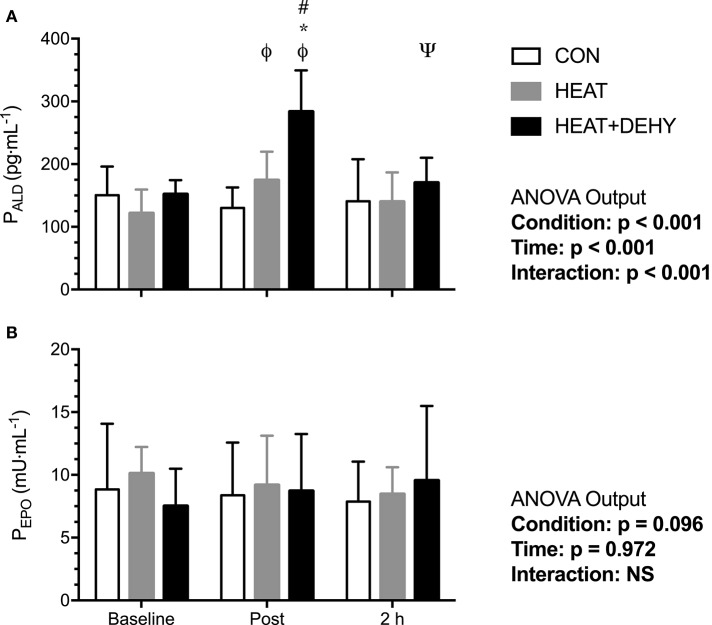
The effect of heat stress and hydration status on plasma aldosterone concentration (P_ALD_, all *n* = 8; **A**) and erythropoietin concentration (P_EPO_; **B**) before, immediately after, and 2-h after the 90-min exercise in CON (all *n* = 8), HEAT (all *n* = 5), and HEAT+DEHY (all *n* = 6) condition. Statistical significance (ANOVA Output) is illustrated. Data presented are mean ± SD. ^Φ^Denotes significantly different (*p* < 0.05) to baseline. ^Ψ^Denotes significantly different to immediately post-exercise measurement. ^*^Denotes significantly different than CON. ^#^Denotes significantly different to HEAT.

Aldosterone concentrations (*n* = 8) were similar between conditions at baseline (all *p* ≥ 0.145). Aldosterone was unchanged across exercise and at the measured (2 h) recovery point in CON (all *p* ≥ 0.472), whereas it increased by 41% after exercise in HEAT ([9, 73]; *p* = 0.010), and by 82% in HEAT+DEHY ([56, 108]; *p* < 0.001; Figure 8A). When comparing conditions, post-exercise ALD concentration tended to be 28% higher by virtue of heat stress ([2, 58]; *p* = 0.071), and 62% higher again by adding dehydration ([39, 86]; *p* < 0.001). These effects were no longer evident at 2 h of recovery (*p* ≥ 0.163 between conditions).

#### Erythropoietin (EPO; Figure 8B)

Erythropoietin concentrations did not change significantly with exercise in CON (*n* = 8), HEAT (*n* = 5), or DEHY (*n* = 6; Figure 8B).

#### Aldosterone-mediated PVE (Figure 9)

**Figure 9:**
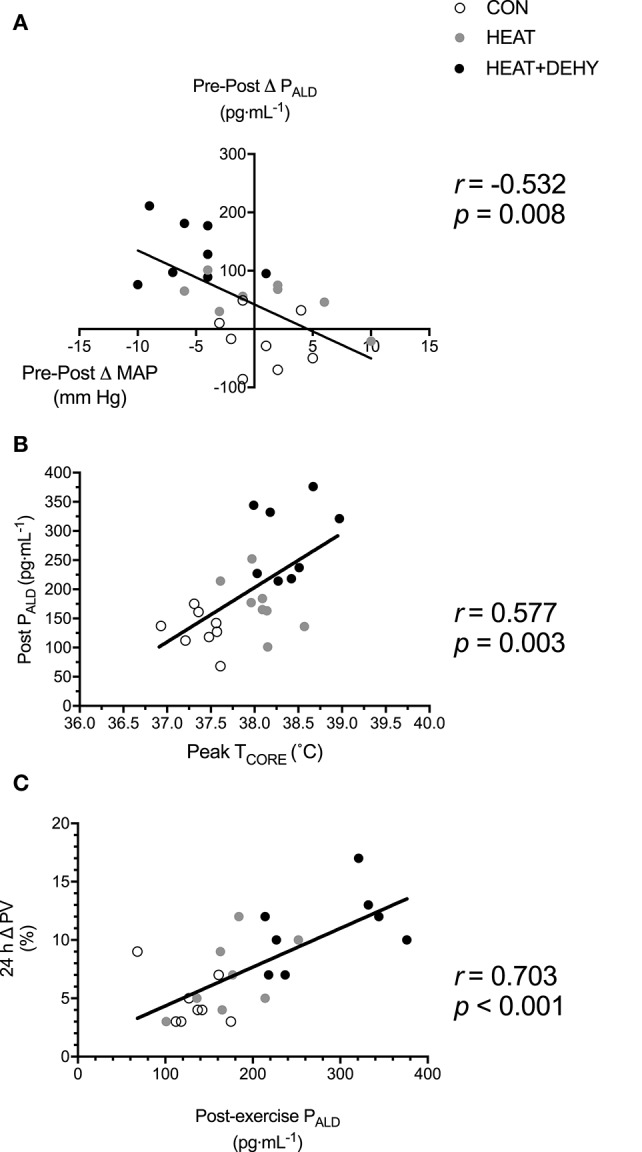
Potential mechanisms of increased aldosterone concentration and its role in mediating the post-exercise hypervolemic response. Illustrated is the relation between the change in MAP immediately after exercise **(A)**, and peak T_CORE_ in exercise **(B)**, and P_ALD_ immediately after exercise (*n* = 8); and the relation between post-exercise P_ALD_ concentration and the 24-h change in PV **(C)**.

The association between cardiovascular (Δ MAP; Figure 9A) and thermal strain (peak T_CORE_; Figure 9B) and ALD concentration were examined to identify potential thresholds and mechanisms of the ALD response. In turn, the relation between the ALD response and PVE was examined to investigate potential mechanisms for exercise-induced hypervolemia (Figure 9C). Larger reductions in MAP and larger rises in T_CORE_ across exercise were strongly associated with post-exercise ALD concentrations, which was strongly predictive of PVE at 24 h.

## Discussion

This is the first study, to our knowledge, to investigate the additive roles of low-intensity, orthostatically-stressful exercise, heat, and dehydration on early cardiovascular outcomes. The main novel findings were that: (i) the addition of these separate stressors combine to augment short-term (24 h) cardiovascular and hematological outcomes, particularly PEH and PVE; (ii) stress-induced strain, particularly the magnitude of T_CORE_ elevation, closely predicts these outcomes; and (iii) within the first 24 h, PEH at least partly facilitates PVE at 24 h. Acute outcomes, such as PEH and PVE are important in their own right, but must also be considered in the context as a precursor for short- and long-term cardiovascular and hematological adaptations. Collectively, these findings indicate that manipulating the strain elicited during an exercise/conditioning bout could facilitate short-term (24 h) outcomes that may aid in exercise performance or cardiovascular health.

### Post-exercise hypotension

Low-intensity exercise provides a widely accessible means of meeting physical activity guidelines, but falls below the threshold required to meaningfully impact some important cardiovascular health parameters. In the current study, low-intensity exercise alone did not elicit a significant drop in BP at 24 h (Figure 3). This supports findings of Forjaz et al. (2004), who found no reduction in MAP 22 h after normotensive individuals cycled for 45 min at 30% $\overset{\cdot }{\text{V}}$O_2 MAX_, but significant reductions in BP after 50 and 75% $\overset{\cdot }{\text{V}}$_2 MAX_. Similarly, Wallace et al. (1999) demonstrated no PEH 22 h after intermittent walking (50% $\overset{\cdot }{\text{V}}$O_2 MAX_) in normotensive individuals. Such data indicate that low-intensity exercise, particularly in normotensive young individuals, may be insufficient to elicit acute BP responses. Acute resistance exercise may also be insufficient in this respect (O'Connor et al., 1993), or more pronounced with large muscle mass exercise and those with hypertension (Casonatto et al., 2016). Even with resistance exercise (60% 1RM) eliciting sufficient stimulus for acute PEH, this may not eventuate over a longer period (i.e., 12 week; Moraes et al., 2012). Thus, higher intensity exercise, or a conditioning stimulus that provides a larger acute perturbation may be required to acquire the long-term (chronic) anti-hypertensive effects of exercise (Quinn, 2000; Thompson et al., 2001; Liu et al., 2012).

Adding heat stress to low-intensity exercise did not consistently affect BP. Elevated core and skin temperatures associated with exogenous heat stress (Table 1) were expected to elicit a greater degree of exercise-induced sympathetic activity (Ray and Gracey, 1997), and subsequent post-exercise residual systemic vasodilation, and thus greater baroreflex resetting and associated sympatho-inhibition (Halliwill et al., 2013). The combined stressors could conceivably also have larger transient effect on oxidative stress and the ensuing balance of oxidative stimuli and anti-oxidative capacity. We hypothesized that this would ultimately manifest in larger PEH at 24 h. Whilst systolic pressure tended to be reduced at 24 h after exercise in the heat (*p* = 0.077), diastolic and mean arterial pressures were not (Figure 3). Post-exercise hypotension is a complex and multifactorial response involving both peripheral and central mechanisms (Halliwill et al., 2013; Graham et al., 2016). Halliwill et al. (2013) suggest that the situational occurrence of hyperthermia may modulate the hypotensive response, however the additional thermal strain incurred in the present study did not have measurable effect. Elevated T_CORE_ in recovery may (Kenny and Niedre, 2002) or may not (Jones et al., 2007) affect immediate PEH (≤30 min after exercise), but this is likely modulated by the preceding exercise intensity (Kenny and Niedre, 2002; Kenny et al., 2006; Jones et al., 2007). Due to the self-determined intensity of the calisthenics, participants may have attenuated the intensity of contractions in the face of a higher cardiovascular and thermal strain, and thus provided insufficient additional stimulus for PEH. While self-pacing is ecologically valid, it may be instructive to use matched paced exercise to provide a more complete mechanistic understanding.

Blood pressure was significantly reduced at 24 h only when modest (3% BM; P_OSM_ ~290 mOsmol·kg^−1^) dehydration was additively imposed on heat stress and low-intensity exercise (Figure 3). Furthermore, every participant demonstrated a reduction in MAP at 24 h when exposed to this combination of stressors (Figure 3), and to a meaningful extent, of 4 mm Hg (Chobanian et al., 2003). Acute dehydration augments physiological strain in multiple systems (Montain and Coyle, 1992b; Sawka et al., 1992; Gonzalez-Alonso, 1998; Gonzalez-Alonso et al., 1999), particularly via exacerbated tissue temperatures and cardiovascular strain in exercise (Montain and Coyle, 1992b; Sawka et al., 1992). This exacerbated strain facilitated PEH in the current study, presumably due at least in part to the thermal and cardiovascular strain elicited in response to this combination of stressors (Figure 4). The strain profile may therefore be as important as the work done, in determining the acute hypotensive response to exercise (Jones et al., 2007). If so, varied methods of modulating the strain induced by exercise (e.g., transient dehydration, ischemia, gravitational) may provide feasible means of eliciting (specific) short- and longer-term adaptations when not accessible via traditional and well-justified means (esp. moderate- and higher-intensity exercise).

Unfortunately, hydration status is sparsely reported in studies investigating PEH (Endo et al., 2012), likely due its complex association with muscular activity, heat production, and thermoregulatory responses. Fluid intake has been shown to abolish PEH in healthy sedentary males in a temperate environment (Endo et al., 2012), and in trained males in a hot (42°C and 20% RH) environment (Gagnon et al., 2012). Both these aforementioned studies demonstrated PEH in the 60–90 min after moderate exercise (1 h at 60% HRR, and 2 h at 120W, respectively) and modest dehydration (~1 and 2.5%, respectively). Further, the magnitude of PEH in early recovery was comparable to that of the current study (−5 mm Hg immediately after exercise; Table 3), but those studies did not report BP for the following day. Alterations in cardiac baroreflex sensitivity may partly explain the observed early-phase PEH in all three studies, as prevention of dehydration (by saline infusion or drinking) during exercise minimizes PEH and abolishes reductions to cardiac baroreflex sensitivity (Charkoudian et al., 2003). Furthermore, when coupled with higher tissue temperatures (Table 1 and Figure 4), and the subsequent cutaneous vasodilation, dehydration may facilitate larger PEH via both inhibition of central vasoconstrictor output and sustained post-exercise vasodilation of active muscle beds (i.e., via central and peripheral mechanisms; Halliwill et al., 2013).

### Hypervolemia

Plasma volume was elevated by 5% at 24 h with calisthenics alone (Figure 5). This magnitude of PVE was unexpected considering its similarity to that obtained from higher-intensity exercise models (Gillen et al., 1994; Yang et al., 1998; Nagashima et al., 1999, 2000), and the fact that these participants were regularly physically active. The dynamic and orthostatically stressful nature of this exercise may therefore partly explain the observed PVE. Rapid PV reductions during exercise are attributed partly to increased capillary hydrostatic pressure and muscle tonicity (Lundvall et al., 1972), muscular activity *per se* (Sjogaard and Saltin, 1982), and a more upright posture (Nagashima et al., 2000). Thus, sustained isometric contractions, adjustments in posture, and dynamic movement sequences using most muscle groups in the current exercise protocol may facilitate a modest PVE despite its relatively low intensity. Plasma volume changes in the current study must also be considered in the context of potential limitations. First, the 10-min rest period prior to capillary sampling was insufficient to allow full stabilization, but was consistent for each individual between conditions, thus no systematic bias is expected. Second, we cannot discount possible confounding by incomplete washout between our conditions (Gillen et al., 1991), but any such effects would also lead to under- rather than over-estimation of hypervolemic effects of exercise or the additional stressors.

Fluid-regulatory hormonal responses to exercise are important mediators of PVE following exercise and heat exposure, and are influenced by exercise intensity and posture (Francesconi et al., 1983a; Ray et al., 1990; Gillen et al., 1991). The 5% increase in PV in CON was not accompanied by an increase in aldosterone (ALD; Figure 8A). Elevated ALD following exercise is involved in renal reabsorption of sodium and coupled reabsorption of water and subsequent PVE (Wade et al., 1985). Longer duration submaximal exercise elicits larger increases in ALD than does maximal exercise (Costill et al., 1976; Wade et al., 1987) but may have a threshold dependence (~60% $\overset{\cdot }{\text{V}}$O_2MAX_; Tanaka et al., 1986). The current study is limited in its assessment of aldosterone-mediated effects on 24-h PVE given the lack of plasma osmolality (P_OSM_) measurements at this time point. Subsequent research should further determine the mechanisms of hypervolemia in response to different strain profiles utilizing a comprehensive assessment of body fluid regulation. Thus, the PVE in CON may therefore have been mediated by mechanisms other than those measured in the current study, including increased content of albumin due to increased lymphatic return, synthesis and decreased transcapillary escape (Gillen et al., 1991; Haskell et al., 1997; Yang et al., 1998).

Heat stress did not additively contribute to PVE (at 24 h) following low-intensity exercise (Figure 5A), despite increasing PV loss during exercise (Figure 5B). Exercise intensity (Nadel et al., 1979) and environmental temperature (Nadel et al., 1979; Maw et al., 1998) affect the PV loss during exercise, such that PV losses are larger in higher ambient temperatures regardless of hydration state (Kenefick et al., 2014). A more pronounced rise in T_CORE_ (Table 1), and concomitant cutaneous vasodilation was expected to elevate capillary hydrostatic pressure, promoting net filtration and therefore augment the PV losses (Starling, 1896; Saltin, 1964; Senay, 1975). However, this larger loss during exercise was not compensated for by a larger rebound hypervolemia. Environmental conditions are also known to affect ALD secretion (Davies et al., 1981), with the increase in T_CORE_ modulating this effect (Moller et al., 1989; Auernhammer and Strasburger, 1995; Wright et al., 2010). Despite a ~40% increase in ALD concentration in HEAT, this did not additively effect PVE to measurable extent; possibly due to the modest thermal strain (~38°C) coupled with the low intensity of exercise.

The combination of exercise, heat stress and dehydration resulted in the largest PVE (Figure 5). Dehydration can impair endurance exercise performance, due in part to increased tissue temperatures and cardiovascular strain (Sawka et al., 1992, 2011; Montain and Coyle, 1992a). These factors are also critically involved in acute hypervolemia (see Figure 2 in Akerman et al., 2016), however the obligatory mechanisms (if any) are largely unclear. Skin temperature was equivalently high in HEAT and HEAT+DEHY (Table 1), thereby rendering it as being unlikely in the mediation of PVE. These findings support those of Armstrong et al. (1997) and Gonzalez-Alonso et al. (1997) and indicate that hyperthermia and dehydration equally but not additively affect T_SKIN_ in these conditions. These findings thereby also support the case for T_CORE_ as a primary and more important modulator of PVE (Maw et al., 2000).

Fluid regulatory hormones are sensitive to changes in body water content and very likely contributed to the observed PVE in HEAT+DEHY. Hypohydration elicits larger increases in plasma renin activity (PRA) and ALD during light exercise in hot environments (Francesconi et al., 1983b; Maresh et al., 2004), whereas such exercise in a temperate environment is insufficient to stimulate these hormones (Finberg et al., 1974; Francesconi et al., 1983b). Furthermore, Francesconi et al. (1983b) also demonstrate an additive effect of heat stress on low-intensity exercise, and more-so with dehydration. The additive effect of dehydration in the current study was also a similar magnitude as Francesconi et al. (1983b), with dehydration approximately doubling the effect of heat alone on ALD concentration. Finberg et al. (1974) also found an association between PRA and cardiovascular strain in light exercise, which were attenuated when euhydration was maintained. Unlike Finberg et al. (1974), the increase in ALD in the current study was associated with the degree of thermal as well as cardiovascular strain experienced (Figure 9). It therefore appears that whilst ALD concentration, and its mediating effect on PVE (Figure 9; Nagashima et al., 1999) may be related most closely with thermal strain (i.e., T_CORE_), other mechanisms of PVE may also contribute.

Heat stress and physical activity act additively to vasoconstrict the splanchnic and renal beds (Rowell et al., 1968). The combination of these stressors may therefore collective facilitate the erythropoietic response to exercise (Sawka et al., 2000), but whether this occurs acutely has yet to be determined. We therefore hypothesized that the three stressors examined here would attenuate splanchnic and renal blood flow and might thus promote an erythropoietic response. However, plasma erythropoietin (EPO) was unchanged in all conditions (Figure 8B). The stimulus may therefore have been insufficient to reduce renal PO_2_, the primary stimulus for EPO release. Plasma volume expansion without an accompanying erythropoeitic stimulation may be beneficial in untrained people, but its functional relevance in highly endurance-trained people is less clear (Hopper et al., 1988). Whereas, PVE in conjunction with red cell volume (RCV) expansion is functionally relevant in trained individuals (Heinicke et al., 2001; Lundby et al., 2016). It is therefore valuable to know what exercise-related stimuli would expand RCV. Elevated EPO concentrations are observed in response to reductions in central venous pressure (CVP; Gunga et al., 1996a), thermal stimuli (Gunga et al., 1996b), and hypovolemia (Szygula et al., 1995), independent of hypoxia (Kirsch et al., 2005). Yet, EPO was not elevated by any of the stimuli used in this study, so their erythropoietic potential remains unclear.

### PEH and PVE

The causal relation between PEH and subsequent PVE is complex and not fully understood. In the current study, the two phenomena were strongly associated, but also showed some independence, as has been observed previously (Holtzhausen and Noakes, 1995; Graham et al., 2016). For instance, PVE was consistently demonstrated in CON and HEAT despite no significant change in MAP. Whilst CVP unloading, and thus some aspect of post-exercise BP reduction must arise as a stimulus for PVE (Nagashima et al., 1999; Hayes et al., 2000), evidently PVE can also occur via other mechanisms.

Arterial pressure is generated by cardiac output and systemic vascular resistance. For PVE to occur with a simultaneous reduction in arterial BP, then presumably arterial or arteriolar resistance must decrease, even if the PVE is accommodated mostly within less resistive venous vessels. With the combination of all three stressors, a rise in cardiac output and T_SKIN_ would facilitate higher skin blood flow and volume, and concomitant reduction in CVP. Atrial natriuretic peptide (ANP) release would be suppressed in these circumstances, thereby enhancing renin-angiotensin-aldosterone system (RAAS) activity. Considering that ALD is slow to act (Collins, 1966; Morel and Doucet, 1986; Nagashima et al., 2001), ANP suppression may also contribute to the rise in PV. Following a rise in PV, any ANP-mediated anti-diuresis may be inefficient in the face of a maximally stimulated RAAS (Altenkirch et al., 1990). However, the vasodilatory effects of ANP (Faber et al., 1988) may conceivably contribute to the occurrence of PEH, thus promoting a reduction in arterial pressure without an efficient anti-diuretic response. Histamine-mediated post-exercise vasodilation (Lockwood et al., 2005; McCord et al., 2006; Barrett-O'Keefe et al., 2013) and alterations in baroreflex control (Halliwill et al., 1996) appear to primarily facilitate PEH independent of changes in BV (Gillen et al., 1994). Post-exercise hypotension is therefore a complex phenomenon involving peripheral and central mechanisms, and its interaction with vascular volumes in still incompletely understood. Hypotheses have postulated to explain apparent links between arterial pressure, volume expansion and sympatho-vagal balance (e.g., Yun et al., 2005) but they are still tentative and warrant further examination.

### Perspectives and summary

The results of the current study indicate the importance and cumulative role of discrete stressors (low-intensity exercise, heat, dehydration) in stimulating acute cardiovascular outcomes. Of note, PEH at 24 h after exercise was affected primarily by the addition of dehydration, with the largest response (and all participants responding) when all stressors were combined, and the largest strain produced. Furthermore, PVE at 24 h after exercise was also largest when exposed to all stressors. The exacerbated strain, particularly thermal, facilitated an increase in ALD, which mediated the rise in PV and was associated with the magnitude of PEH. The current study provided associations (i.e., non-causal) between additive stressors and outcome measures. Future research should investigate dose-response relations between mediating factors of acute PEH and PVE, particularly the occurrence of incremental hyperthermia and these outcomes. The same acute outcomes may also be possible without the imposition of dehydration, but instead increasing T_CORE_ by other means (e.g., more intense exercise or thermal stress). Whilst this is a reasonable assumption, not only does dehydration exacerbate the thermal strain of exercise in the heat (with no/low airflow at least), its independent effects may also provide a conditioning stimulus as detailed above. However, the role of dehydration in the context of acute conditioning is rarely considered. Future research should aim to identify the means by which dehydration may aid (or hinder) adaptation to exercise, rather than its well-established effects on performance acutely. In particular, the additive roles of individual stressors should be examined in a more extensive assessment; including (i) other known cardiovascular (CVP, SV, $\overset{\cdot }{\text{Q}}$, TPR) and fluid regulatory (AVP, ANP, ALB) modulators of PEH and PVE, and (ii) the recovery kinetics of body fluid dynamics (across minutes to days), as these are seldom considered (Luttrell and Halliwill, 2015).

Acute periods of high strain, or the resultant outcomes may also be unwarranted in particular individuals. Acute PEH, whilst beneficial to most individuals, would be unwarranted in individuals with hypotension or postural orthostatic tachycardia syndrome (POTS). Similarly, dehydration is unlikely to be recommended for clinical populations, as recurrent dehydration (Clark et al., 2016), or chronic stimulation of fluid- and sodium-conserving hormones may contribute to disease progression (Thornton, 2010). However, in those undergoing conditioning periods (e.g., exercise training), the exacerbated strain may (Garrett et al., 2014) or may not (Neal et al., 2016) elicit larger changes in short-term cardiovascular and hematological adaptive processes. Equally, high levels of hypohydration may also interfere with muscle metabolic control, protein synthesis and hypothalamic adaptations to heat acclimation (Schliess et al., 2006; Schwimmer et al., 2006). Thus, whilst periods of sustained hypohydration are likely unwarranted in certain clinical populations, it is currently unclear whether transient mild dehydration (and the strain therein) plays a substantial role in the process of exercise-associated adaptation (Akerman et al., 2016).

In conclusion, acute cardiovascular and hematological outcomes following exercise therefore appear to be (at least partly) determined by the magnitude of strain induced in the conditioning stimulus. The addition of multiple independent stressors to a low-intensity exercise may exacerbate the strain, and thus the potential adaptive stimulus. This added strain might be useful when exercise performance *per se* is not the primary outcome; in those unable to complete traditional exercise modes (e.g., elderly or injured); are anticipating a novel acute stress (e.g., before a heat wave or surgery); or avoiding intense exercise (e.g., tapering phase before competition for athletes). Future research should investigate whether this complementary response is evident in other modes and intensities of exercise, and what facilitates such adaptive responses.

## Author contributions

AA, SL, and JC contributed to the conception and design of the study, acquisition, analysis and interpretation of the data, drafted, revised and approved the final version of the manuscript. RK contributed to analyzing the data (ELISA analysis) and reviewed and approved the final version of the manuscript.

### Conflict of interest statement

The authors declare that the research was conducted in the absence of any commercial or financial relationships that could be construed as a potential conflict of interest.

## Supplementary material

Supplementary Figure 1 Example progression (from left to right, starting top left corner) of exercises during the 70-min callisthenics period of the exercise protocol.

Supplementary Figure 2 The effect of heat stress and hydration status on the systolic (sBP; **A**), diastolic (dBP, **B**) and mean arterial blood pressure (MAP, **C**) response to the 90-min exercise condition, and in recovery. Data presented are group mean with individual values overlaid. m denotes male participant. For statistical significance please refer to Figure 5 in the Manuscript.
