# Supplementary figures and images for: Heat and Dehydration Additively Enhance Cardiovascular Outcomes following Orthostatically-Stressful Calisthenics Exercise
Source: Front Physiol. 2017 Oct 9;8:756. doi: 10.3389/fphys.2017.00756 (PMC5640974; doi:10.3389/fphys.2017.00756)

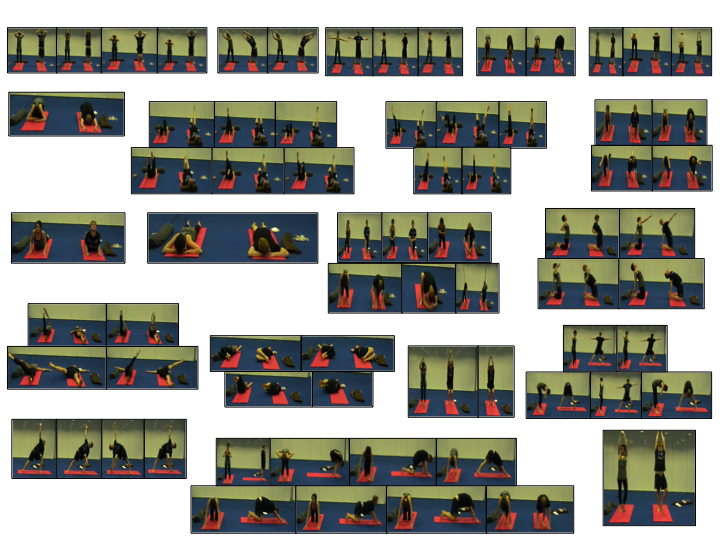

Supplement: Supplementary Figure 1 — Example progression (from left to right, starting top left corner) of exercises during the 70-min callisthenics period of the exercise protocol. [file Image1.png]
