# Supplementary material for: Heat and Dehydration Additively Enhance Cardiovascular Outcomes following Orthostatically-Stressful Calisthenics Exercise
Source: Front Physiol. 2017 Oct 9;8:756. doi: 10.3389/fphys.2017.00756 (PMC5640974; doi:10.3389/fphys.2017.00756)
Supplement: Supplementary file 3 [file DataSheet1.docx]

**Callisthenics Protocol**

The callisthenics protocol is described below. The common name of each posture/movement is given in **bold**, and Sanskrit (where appropriate) name given in ***bold italics***. A brief description of the posture/movement is also provided. The video for the callisthenics protocols is hosted at: <https://www.youtube.com/watch?v=FxMdPDUk8Fo>

**Standing**

**Mountain Breathing and posture - *Tadasana***

**Mountain - *Tadasana***

Breathing and Stretching – 3 rounds of stretching onto toes and breathing. Held for 1 – 2 breaths.

**Mountain - *Tadasana***

Explosive movement for stretches – dropping into seated position (1/4 squat – held for 1-2 breaths), and exploding onto toes. 2 rounds.

**Swaying palm Tree pose - *Tiraka Tadasana***

Side stretches (4 rounds per side of the body).

**Waist rotating pose - *Kati Chakrasana***

Rotating twist through the abdomen. 3 rounds per side of the body

**Double angle pose - *Dwikonasana***

Forward bend shoulder stretch. 3-5 rounds.

**Forward bend - *Pada Hastasana***

Standing forward bend. 3 rounds

**Supine Posture**

**Cycling - Pada Sanchalanasana**

One leg cycling for abdominal strength, 3 to 5 rounds per leg.

Reversed cycling motion, 3 to 5 rounds per leg.

3 deep breaths from the lower abdomen.

**Cycling - *Pada Sanchalanasana***

Double leg cycling (simultaneously) forwards

Count of 10 revolutions.

Double leg cycling (simultaneously) backwards. Count of 10 revolutions.

Abdominal breathing 3 deep breaths from the lower abdomen.

**Leg Rotations - *Chakra Padasana***

Clockwise one leg hip circles. 5 revolutions

Anti-clockwise one leg hip circles. 5 revolutions

3 deep breaths from the lower abdomen.

**Leg Rotations - *Chakra Padasana***

Clockwise double leg hip circles. 3 revolutions

Anti-clockwise double leg hip circles. 3 revolutions

3 deep breaths from lower abdomen

Single leg raises. 5 rounds per leg.

**On hands and knees**

**Cat pose - *Marjariasana***

Cat posture (on all fours).

Rounding and arching the back with breaths in and out respectively. 3 to 5 breaths.

***Marjariasana variations***

Balancing cat, alternating lifting opposite arms and legs (i.e., raise left arm, right leg; or right arm, and left leg). 3 to 5 rounds, holding for 1 to 2 breaths at the balancing point.

**Throughout hands and knees positions**

**Position of the hare – *Shashankasana***

Sitting on heels for relaxing posture between progressions.

**Prone position**

**Cobra - *Bhujangasana***

Lower back stretch. Hands under shoulders, arching the back whilst keeping legs together and elbows against the side of body (off the floor). Holding top posture for 1 to 2 breaths. 3 to 5 rounds.

**Sphinx asana posture**

Upper back stretch. Keeping arms on floor ahead of you, and raising the head and upper back. 3 to 5 rounds.

**Throughout prone positions**

**Reversed corpse pose – *Advasana***

Head on back of hands facing to the side for relaxing posture between progressions.

**Combination of postures**

**Equestrian pose - *Ashwa Sanchalanasana***

Hip stretches. Holding each progression for 2 to 3 seconds. 1 round on each leg (all progressions on one leg before progressing to opposite leg).

**Equestrian pose variations - *Ashwa Sanchalanasana***

First progression hand on knee and forward to rest weight on front leg.

Second progression, lying body on front knee.

Third progression, hands by feet and weight inside the hip.

Fourth progression, forearms onto floor and body to floor.

Fifth level, hip and side stretch with one hand to floor, one to the ceiling.

**Camel posture - *Ardha Ushtraasna***

Back bend in kneeling position. Sitting on heels between progressions.

First progression, hand on back and hip pushed forward

Second progression, one hand on heel and hip pushed forward. Other arm forwards and looking at fingers Hold for 2 to 3 seconds on each side.

Third progression, one hand to heel and hip pushed forward. Shoulders aligned. Hold for 2 to 3 seconds on each side.

Final progression, both hands to heel. Hips pushed forward and dropping head back.

Seated on heels for relaxation posture afterwards.

**Hand to toe pose - *Supta Padangusthasnana***

Complete all progressions on one leg before progressing to next leg.

Hamstring stretches. Lying on back with a strap or band around one foot, ends being held. Hold leg in the air, stretching hamstring. Hold for 3 to 5 seconds.

Second progression, bring foot closer to face.

Third progression, hold strap in same hand (to the foot in air). Opposite arm at shoulder height, palm down. Let leg drop away from the body.

Fourth progression, hold strap in opposite hand (to the foot in air). Other arm at shoulder height, pal down. Let leg drop across the body.

**Sleeping abdominal stretch pose - *Supta Udarakarshanasana***

Lying on back with feet by buttocks. Arms behind head, feet and legs dropping onto one-another, head turns in opposite direction. Hold for 5 seconds on each side.

Progression involves the same movement, but the legs and feet off the floor at a right angle. Hold for 5 seconds on each side.

**Standing sequences**

**Dynamic energy pose - *Druta Utkatasana***

Hands interlaced above the head, elbows by ears. Drop into squat (90˚) whilst keeping body (and back straight). 4 rounds.

**Warrior posture - *Virabhadrasana***

One foot facing forward, one side on. Forward lunge side-on, facing forward. Hold for 3 to 5 seconds. 2 to 3 rounds per side.

Forward bend between sides for 1 to 2 breaths.

**Triangle pose - *Trikonasana***

Same starting posture as warrior, but forearm on front bent leg, and other arm towards ceiling. Hold for 3 to 5 seconds on each side.

Progression to palm on the inside of front leg, before straightening both legs and other arm to ceiling. Hold for 3 to 5 seconds on each side.

Forward bend between progressions for 1 to 2 breaths.

**Sun salutations moving sequence (4 versions)- *Surya Namaskar***

Hold each separate sequence/movement for 1 breath.

1^st^ version - Starting at front of mat. Stretch up, followed by forward bend with hands on hips. Hands to either side of feet, and step backwards and dropping knee to ground. Stretching through front hip. Front foot moves backwards in line with back foot, and body moves into a deep V (or downward facing dog). Drop down to hands and knees, and into cat position. Arch and extend on breaths 3 times. Pushing onto knees, stepping one leg forward into lunge with hands beside feet. Other leg comes forward into forward bend, and stretch up. 2 rounds of this version.

2^nd^ version - Starting at front of mat. Stretch up, followed by forward bend with hands on hips. Hands to either side of feet, and step backwards and dropping knee to ground. Stretching through front hip. Front foot moves backwards in line with back foot, and body moves into a deep V (or downward facing dog). Drop down to hands and knees, and into cat position. Arch and extend on breaths 3 times. Toes curling back and pushing into deep V, stepping one leg forward into lunge with hands beside feet. Other leg comes forward into forward bend, and stretch up. 2 rounds of this version.

3^rd^ version - Starting at front of mat. Stretch up, followed by forward bend with hands on hips. Hands to either side of feet, and step backwards and dropping knee to ground. Stretching through front hip. Front foot moves backwards in line with back foot, and body moves into a deep V (or downward facing dog). Knees drop down followed by chin, and then chest in a swooping motion and ending in Cobra posture. Toes curling back and pushing into deep V, stepping one leg forward into lunge with hands beside feet. Other leg comes forward into forward bend, and stretch up. 2 rounds of this version.

4^th^ version – Same as 2nd version but hold each posture for 2 breaths.

**Tree (balancing) posture - *Eka Pada Pranamasana***

First progression balancing on one foot and toes of other foot (heel against calf). Arms raised above the head. Hold for 5 seconds on each leg.

Second progression balancing on one foot. Sole of the other foot on the dominant calf. Arms raised above the head. Hold for 5 seconds on each leg.
